# Silymarin Protects against Acute Liver Injury Induced by Acetaminophen by Downregulating the Expression and Activity of the CYP2E1 Enzyme

**DOI:** 10.3390/molecules27248855

**Published:** 2022-12-13

**Authors:** Weipei Yang, Zhongxu Liang, Chengming Wen, Xuehua Jiang, Ling Wang

**Affiliations:** Key Laboratory of Drug-Targeting and Drug Delivery System of the Education Ministry, Department of Clinical Pharmacy and Pharmacy Administration, West China School of Pharmacy, Sichuan University, Chengdu 610041, China

**Keywords:** silymarin, acetaminophen, CYP2E1, acute liver injury

## Abstract

Previous studies have shown that silymarin protects against various types of drug-induced liver injury, but whether the protective mechanism of silymarin against acetaminophen-induced liver injury is related to the CYP2E1 enzyme remains unclear. In this study, we investigated the effect of silymarin on the activity and expression of CYP2E1 in vitro and in vivo. The results of in vitro studies showed that silymarin not only inhibited the activity of CYP2E1 in human and rat liver microsomes but also reduced the expression of CYP2E1 in HepG2 cells. In vivo studies showed that silymarin pretreatment significantly reduced the conversion of chlorzoxazone to its metabolite 6-OH-CLX and significantly increased the t_1/2_, area under the curve (AUC) and mean residence time (MRT) of chlorzoxazone. In addition, silymarin pretreatment significantly inhibited the upregulation of Cyp2e1 expression, reduced the production of 3-cysteinylacetaminophen trifluoroacetic acid salt (APAP-CYS), and restored the liver glutathione level. The results of our study show that silymarin plays an important protective role in the early stage of acetaminophen-induced acute liver injury by reducing the activity and expression of CYP2E1, reducing the generation of toxic metabolites, and alleviating liver injury.

## 1. Introduction

Acetaminophen (APAP), also called paracetamol, is widely used clinically as an antipyretic and analgesic [1]. Therapeutic doses of APAP are usually safe, but when used at an overdose or below the maximum safe dose for prolonged periods of time, APAP can easily lead to acute liver injury [2]. Acute liver injury caused by APAP overdose accounts for approximately 50% of liver injury cases in the US [3], compared to 40–70% in the UK and Europe [4]. Although the rate of APAP-induced of liver injury is low in Asians, approximately 7.3%, this phenomenon cannot be ignored due to the large size of the Asian population [5]. Given the widespread use of APAP and the frequency of associated acute liver injury, we need to take this problem seriously.

When taken at a therapeutic dose, most APAP binds to glucuronide or sulfate to increase its polarity and is in turn excreted in the urine via the kidneys [6]. The rest of APAP is metabolized by cytochrome P450 enzymes, mainly CYP2E1, to a highly reactive toxic substance, N-acetyl-p-benzoquinone imine (NAPQI), which can be detoxified by binding to glutathione and then eliminated through the bile [7]. Excessive intake of APAP alone or in combination with enzyme inducers can lead to increased NAPQI production via APAP metabolism in vivo; APAP then binds to intracellular proteins containing sulfhydryl groups to form APAP-glutathione conjugates when intracellular glutathione is depleted, leading to hepatotoxicity.

N-acetylcysteine (NAC) is currently the only FDA-approved treatment for APAP overdose [8], but NAC has considerable limitations: NAC is only effective for a short period of time after excessive intake of APAP, namely, the metabolic phase of APAP toxicity. NAC can competitively bind to NAPQI and reduce its binding to intracellular proteins to exert therapeutic effects. When liver injury occurs, the efficacy of NAC is very weak; thus, it is important to develop drugs to treat APAP-induced acute liver injury (ALI) [9]. A number of natural products, among which silymarin is a typical example, have been shown to have protective effects against APAP-induced liver injury [8].

Silymarin (SM) is extracted from the dried fruit of the plant Silybum marianum, a member of the Asteraceae family, and includes polyphenols, flavonoids and other compounds [10] with a variety of pharmacological effects, including hepatoprotective, anti-inflammatory, antioxidant, antifibrotic, and other effects [11]. As an antioxidant, SM is used to treat ALI caused by a variety of factors. Previous studies have shown that SM can be protective against liver fibrosis caused by carbon tetrachloride [12]; liver injury caused by exposure to D-galactose/lipopolysaccharide [13], the heavy metal lead [14], aluminum/fructose [15], thioacetamide [16], and valproic acid [17]; and drug-induced liver injury caused by APAP [18] and cisplatin [19].

Given the prevalence of liver injury cases caused by APAP, it is necessary and important to focus on the protective effect of SM on APAP-induced ALI. The metabolism of excessive APAP produces large amounts of NAPQI, which first depletes intracellular glutathione and then binds to mitochondrial proteins, leading to mitochondrial dysfunction and eventually to oxidative stress and ROS production. Next, downstream signals that induce a series of reactions that lead to hepatocyte necrosis are activated [20]. Current reports on the mechanism underlying the hepatoprotective effects of SM have focused on both the antioxidant and the anti-inflammatory aspects [21]. For example, SM treatment improved steatosis induced by a high-fat diet by inhibiting the inflammatory factors TNF-α, IL-6, and IL-1β in mice. In an animal model of cyclosporin A-induced liver and kidney injury, SM reduced the histopathological changes induced by cyclosporine A in a dose-dependent manner, significantly reducing the levels of the oxidative stress markers superoxide dismutase and glutathione in vivo.

Therefore, SM may alleviate APAP-induced ALI through its antioxidant and anti-inflammatory activities. As NAPQI is primarily produced during APAP metabolism via CYP2E1, factors affecting the activity or expression of CYP2E1 may affect APAP hepatotoxicity. The protective effect of SM on APAP-induced ALI has been reported in several publications, but the role of CYP2E1 in this regard is unclear. Thus, the purpose of this study was to investigate the protective effect of SM on APAP-induced ALI based on the activity and expression of the CYP2E1 enzyme.

## 2. Results

### 2.1. Effect of SM on the Activity and Expression of the CYP2E1 Enzyme

#### 2.1.1. Inhibitory Effects of SM on CYP2E1 Activity in HLMs/RLMs

**Figure 1:**
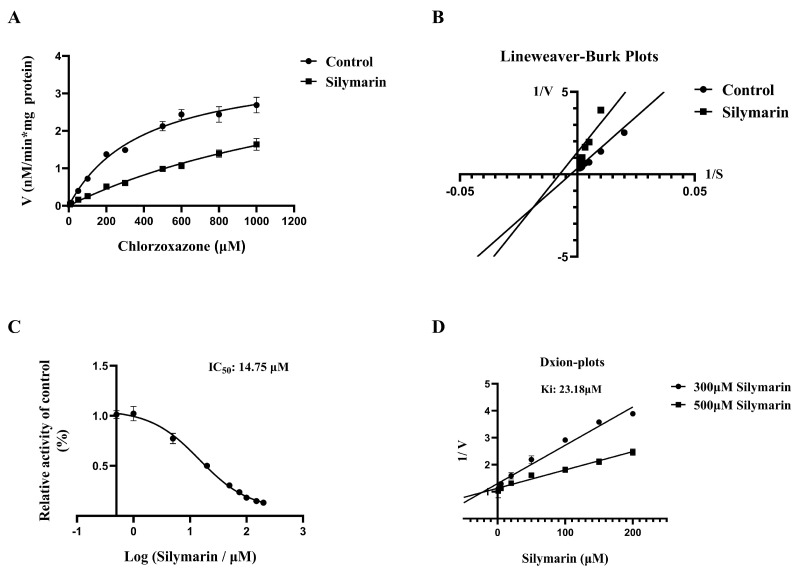
Michaelis-Menten curve (**A**) and Lineweaver-Burk plot (**B**) of CLX in HLMs, (**C**) IC_50_ curve of SM for the inhibition of CLX metabolism in HLMs, and (**D**) Dixon plots of SM in HLMs. The data are presented as the means ± SDs, *n* = 3.

**Figure 2:**
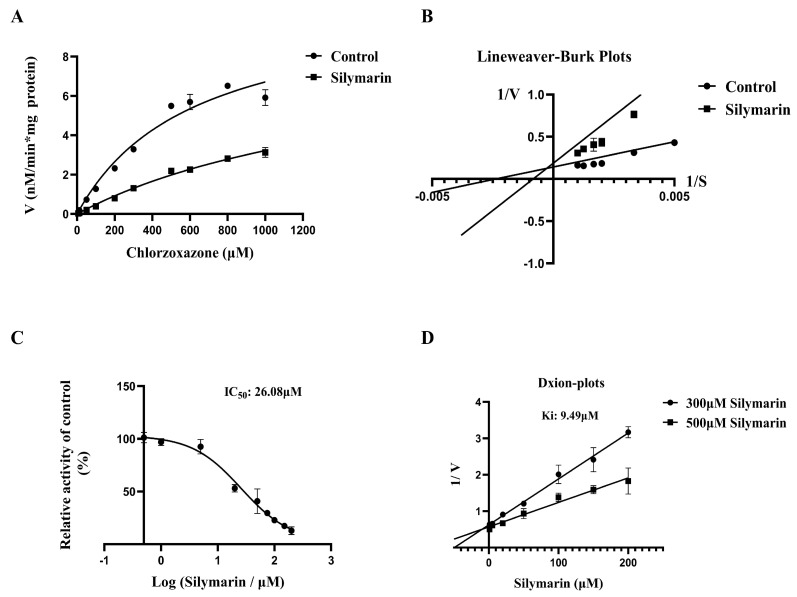
Michaelis-Menten curve (**A**) and Lineweaver-Burk plot (**B**) of CLX in RLMs, (**C**) IC_50_ curve of SM for the inhibition of CLX metabolism in RLMs, and (**D**) Dixon plots of SM in RLMs. The data are presented as the means ± SDs, *n* = 3.

To investigate whether SM affects the catalytic activity of CYP2E1 in RLMs/HLMs, probe assays were conducted with various concentrations of SM. As previously reported, clomethiazole was selected as the positive control for CYP2E1 inhibition. Figure 1A and Figure 2A and Table 1 show that in both HLMs and RLMs, there was no significant change in V_max_ in the SM group compared with the control group, but K_m_ was increased significantly. As shown in the Lineweaver-Burk plots (Figure 1B and Figure 2B), the SM inhibited CYP2E1 metabolic enzyme activity in HLMs/RLMs via mixed-type inhibition.

To further explore the extent to which SM inhibits CYP2E1 activity in HLMs/RLMs, we determined the IC_50_ and K_i_ of SM in HLMs and RLMs. As shown in Figure 1C and Figure 2C, the IC_50_ values of SM in HLMs and RLMs were 14.75 μM and 26.08 μM, respectively. The inhibition data were graphed as Dixon plots (Figure 1D and Figure 2D), and the K_i_ values of SM in HLMs and RLMs were 23.18 μM and 9.49 μM, respectively. The above results indicate that SM can inhibit the metabolic activity of CYP2E1 in HLMs/RLMs, suggesting that SM may affect CYP2E1-mediated drug metabolism (Figure 1 and Figure 2)

#### 2.1.2. Inhibitory Effects of SM on CYP2E1 Activity in Rats

**Figure 3:**
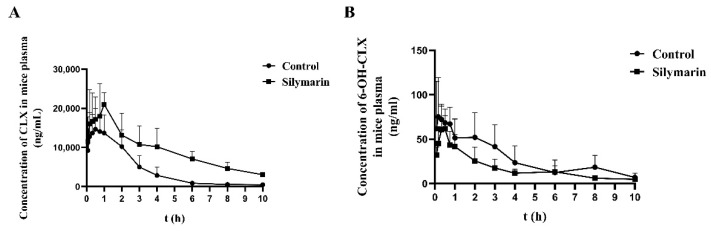
Average plasma concentration–time curves of CLX (**A**) and 6-OH-CLX (**B**) in the solvent control (4% HP-β-CD (*w*/*v*)) and SM (45 mg/kg) groups after a single oral dose of 30 mg/kg CLX. The data are presented as the means ± SDs, *n* = 6.

To further evaluate whether SM affects CYP2E1 activity in rats, we determined the pharmacokinetics of a single oral dose of CLX after 21 days of SM pretreatment. Approximately 90% of CLX is transformed to 6-OH-CLX by CYP2E1 in vivo; thus, CLX was selected as the probe substrate. The average plasma concentration–time curves of CLX and 6-OH-CLX are shown in Figure 3, and the pharmacokinetic parameters of CLX and 6-OH-CLX are summarized in Table 2 and Table 3, respectively. The pharmacokinetic data of CLX showed that the half-life (t_1/2_) and mean residence time (MRT) were significantly prolonged, clearance (CL) was significantly decreased, and the area under the concentration–time curve (AUC_0-t_, AUC_0-∞_) was significantly increased in the SM group compared with the solvent control group. In contrast, the pharmacokinetic data of 6-OH-CLX showed that the AUC_0-t_ and AUC_0-∞_ in the SM group were significantly reduced compared with those in the solvent control group. These results indicate that SM can reduce the enzymatic activity of CYP2E1 in rats and reduce drug metabolism mediated by CYP2E1.

#### 2.1.3. SM Decreased the Expression of CYP2E1 in HepG2 Cells

**Figure 4:**
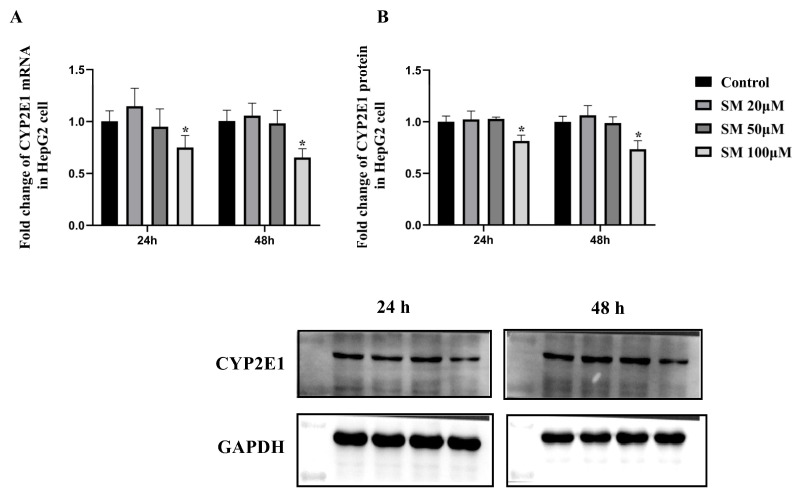
The mRNA expression (**A**) and protein expression (**B**) levels of CYP2E1 in HepG2 cells after 24 h or 48 h of treatment with different concentrations of SM. The mRNA and protein expression levels of CYP2E1 were normalized to those of GAPDH. The data are presented as the means ± SDs, *n* = 3. * *p* < 0.05 compared with the control group.

To assess the effect of SM on the expression of the human CYP2E1 enzyme, we selected different concentrations of SM (20, 50, or 100 μM) to treat HepG2 cells for 24 h or 48 h based on the results of the MTT assay (Supplementary Figure S1). As shown in Figure 4, after 24 h or 48 h of SM treatment, the mRNA and protein expression levels of CYP2E1 in the 20 μM and 50 μM SM groups did not differ significantly compared with those in the control group, whereas the mRNA and protein expression levels of CYP2E1 in the 100 μM SM group were significantly decreased (downregulation of mRNA expression approached 25% and downregulation of protein expression approached 20% after treatment for 24 h; downregulation of both mRNA and protein expression approached 35% after treatment for 48 h), as shown by RT-qPCR and Western blotting. These data indicate that SM can inhibit the expression of human CYP2E1 enzyme at a concentration of 100 μM.

### 2.2. Protective Effect of SM on APAP-Induced ALI

#### 2.2.1. SM Decreased Serum ALT and AST Levels and Restored Liver GSH Levels in Mice

**Figure 5:**
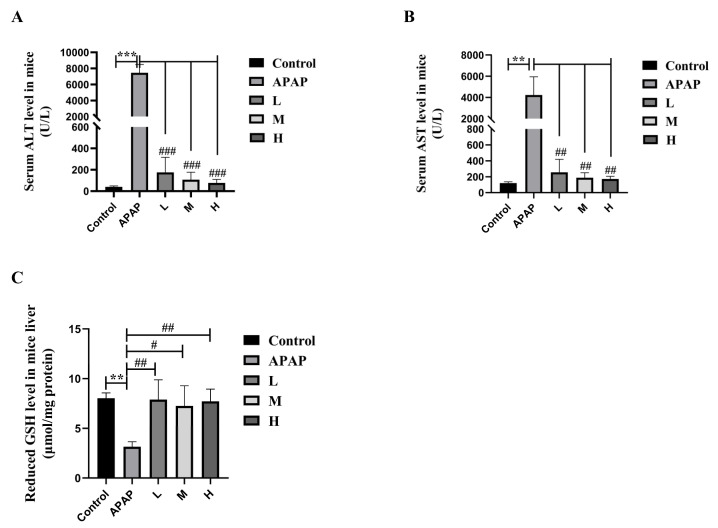
The levels of serum ALT (**A**), AST (**B**), and liver reduced GSH (**C**) in normal (pre-treated with solvent) and ALI (pre-treated with SM) mice. Series dosages of 35, 50, and 65 mg/kg were noted as L, M, and H, respectively. The data are presented as the means ± SDs, *n* = 6. ** *p* < 0.01, *** *p* < 0.001 compared with the control group; # *p* < 0.05, ## *p* < 0.01, ### *p* < 0.001 compared with the APAP group.

To investigate the protective effect of SM on the hepatotoxicity of APAP, serum ALT and AST were selected as biochemical markers to indicate the degree of liver injury. As shown in Figure 5A,B, compared with those in the solvent control group, the levels of serum ALT and AST were significantly increased in the APAP group. However, SM (35, 50, or 65 mg/kg) pretreatment markedly decreased the levels of serum ALT and AST compared with those in the APAP group.

When there is excessive APAP in vivo, a small amount of APAP is converted into NAPQI by CYP2E1. NAPQI can bind to reduced GSH for detoxification. Therefore, we evaluated the protective effect of SM on APAP-induced ALI by measuring the levels of reduced GSH. As shown in Figure 5C, compared with that in the solvent control group, the content of reduced GSH in the APAP group was significantly reduced. However, compared with that in the APAP group, the content of reduced GSH in the liver was restored to almost normal levels in the SM (35, 50, or 65 mg/kg) pretreatment groups. The above results indicate that SM pretreatment can reduce serum ALT/AST and restore the GSH level, thereby alleviating liver injury caused by APAP overdose.

#### 2.2.2. Effects of SM on Plasma Concentrations of APAP and Its Metabolites

**Figure 6:**
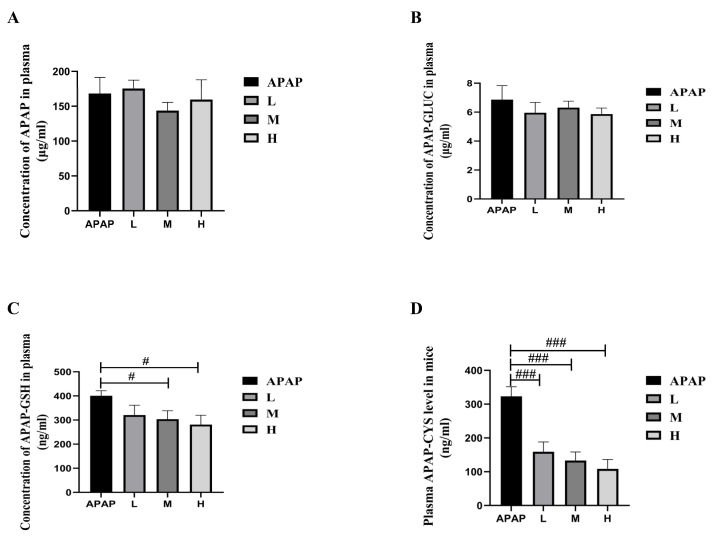
Plasma concentrations of APAP (**A**), APAP-gluc (**B**), APAP-GSH (**C**), and APAP-CYS (**D**) in ALI (pre-treated with SM) mice at 6 h after APAP administration. Series dosages of 35, 50, and 65 mg/kg were noted as L, M, and H, respectively. The data are presented as the means ± SDs, *n* = 6. # *p* < 0.05, ### *p* < 0.001 compared with the APAP group.

In order to investigate the effect of SM on the metabolic process of APAP in vivo, we measured the plasma concentrations of APAP, APAP-gluc, APAP-GSH, and APAP-CYS in ALI mice at 6 h after APAP administration. As the results showed in Figure 6, the plasma concentrations of APAP and APAP-gluc in groups treated with SM (35, 50, or 65 mg/kg) did not change significantly compared with group APAP, indicating that SM has no impact on the glucuronide-mediated metabolism of APAP. In contrast, the levels of APAP-GSH and APAP-CYS in the SM pretreatment group were significantly reduced compared with group APAP, which suggested that the production of NAPQI might be decreased.

#### 2.2.3. SM Suppressed the Upregulation of Cyp2e1 Expression in the Livers of APAP-Induced Mice

**Figure 7:**
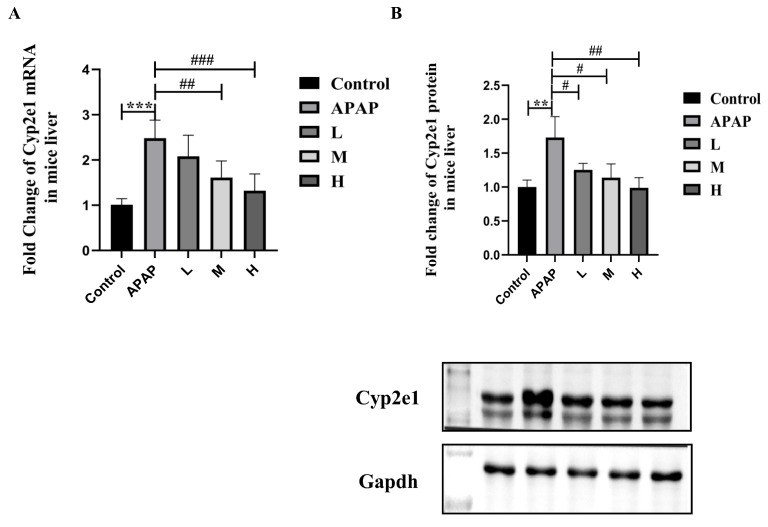
The mRNA expression (**A**) and protein expression (**B**) of Cyp2e1 in normal (pre-treated with solvent) and ALI (pre-treated with SM) mice. Series dosages of 35, 50, and 65 mg/kg were noted as L, M, and H, respectively. The mRNA and protein expression levels of Cyp2e1 were normalized to Gapdh. The data are presented as the means ± SDs, *n* = 6. ** *p* < 0.01, *** *p* < 0.001 compared with the control group; # *p* < 0.05, ## *p* < 0.01, ### *p* < 0.001 compared with the APAP group.

To explore whether SM pretreatment affects the expression of Cyp2e1 in mouse liver tissue, we used RT-qPCR and Western blot analyses to measure the expression of Cyp2e1. After 21 days of pretreatment with low, medium, and high doses of SM, mice in all experimental groups received a single intraperitoneal dose of APAP. As shown in Figure 7, the mRNA and protein expression of Cyp2e1 was significantly upregulated in the APAP group compared with the solvent control group. However, compared with that in the APAP group, the expression of Cyp2e1 in the SM pretreatment group was significantly downregulated in a dose-dependent manner. These data indicated that the expression of Cyp2e1 in the mouse liver was significantly upregulated by APAP overdose but that this tendency toward upregulation was significantly inhibited by SM pretreatment.

#### 2.2.4. SM Ameliorates APAP-Induced Pathological Liver Injury in Mice

**Figure 8:**
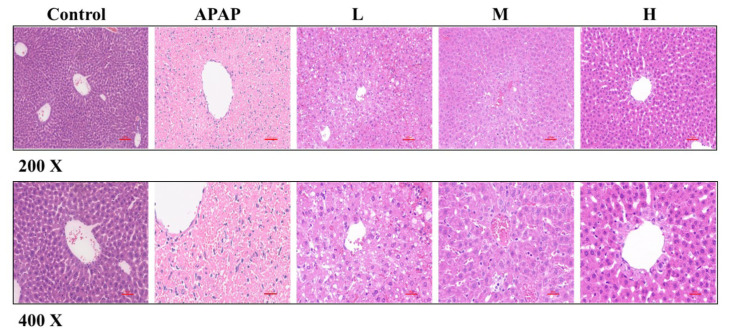
Representative micrographs of H&E-stained liver right lower lobe sections from normal (pre-treated with solvent) and ALI (pre-treated with SM) mice. Series dosages of 35, 50, and 65 mg/kg were noted as L, M, and H, respectively. (200× and 400× magnifications).

To evaluate the protective effects of SM more comprehensively, we performed histopathological analyses to assess the necrosis and apoptosis of hepatocytes. Histological analysis by H&E staining indicated that the hepatic lobule structure was intact and that the liver cords were arranged radially in the solvent control group, while in the APAP group, the hepatic lobule structure was severely damaged, and the hepatocyte arrangement was disordered. There was extensive necrosis of hepatocytes around the central vein, and numerous inflammatory cells had infiltrated the necrotic area. As expected, silymarin (35, 50, or 65 mg/kg) pretreatment alleviated hepatocyte necrosis induced by APAP overdose in mice, and the degree of liver injury reduction was positively correlated with the dose of SM (Figure 8). In conclusion, SM can alleviate liver pathological injury induced by APAP overdose in mice.

## 3. Discussion

In vitro metabolic studies showed that SM inhibited CYP2E1 activity in both RLMs and HLMs. The Lineweaver–Burk plot can provide an intuitive sense of the different forms of enzyme inhibition. For example, inhibitors belong to competitive inhibitors when the two lines intersect on the *y* axis or noncompetitive inhibitors when the two lines intersect on the *x* axis, and uncompetitive inhibitors produce a series of parallel lines with different intercepts on the *y* and *x* axes [22]. However, the plots of SM did not match these three classical inhibition types in both RLMs and HLMs; thus, we speculated it may have a mixed inhibition type. SM showed different inhibitory effects on CYP2E1 activity in HLMs and RLMs, that the IC_50_ values were higher in HLMs whereas the Ki values were higher in RLMs. Generally, IC_50_ value is often affected by the inhibitor concentration, microsomal concentration, and incubation conditions; thus, the IC_50_ values of inhibitors measured in different laboratories may be different and can only be used to roughly assess the inhibitory magnitude of a specific enzyme. On the contrary, Ki values may be less affected by experimental conditions and could be more appropriate for assessing the inhibitory degree of the enzyme than IC_50_ values [23]. Collectedly, we suggested that SM showed a stronger inhibition on CYP2E1 activity in RLMs than HLMs according to the Ki values.

The cell experiments exhibited that 100 μM SM significantly downregulated the mRNA and protein content of CYP2E1 in HepG2 cells, while 20 μM and 50 μM SM had no such effect. Interestingly, the K_i_ value of SM on CYP2E1 in HLMs was 23.18 μM as described in the in vitro metabolic study before. Therefore, together with the results of the cell experiments, we suggested that SM at the concentration lower than 100 μM may have no effect on the CYP2E1 expression but can still impact its metabolic activity and lead to the potential drug–drug interaction in vivo. These effects must be considered when combining SM with drugs metabolized by CYP2E1 in clinical medication.

To further evaluate the effects of SM on CYP2E1 in vivo, we conducted the pharmacokinetics study of CLX in rats. The data showed that SM pretreatment significantly increased the t1/2, MRT, and AUC and decreased the CL of CLX, but had no significant effects on its T_max_, C_max_, and V*d*. These results indicated that SM increased the exposure of CLX mainly by inhibiting its metabolism mediated by CYP2E1 and had no effects on its absorption and distribution. However, the expression of CYP2E1 in the livers of rats was not significantly changed after SM pretreatment (Supplementary Figure S2). We speculated that multiple-dose administration of SM may only reach an effective concentration in normal rats’ liver to inhibit the CYP2E1 activity but not reach an effective concentration to affect its expression, which could be supported by the data shown in Supplementary Materials, Table S4, in that the liver concentration of SM was higher than the Ki value in RLM; moreover, it may exit other specific ways that needed to be confirmed by further studies.

The generation of NAPQI is a key factor leading to liver injury [24]. CYP1A2, CYP3A4, and CYP2E1 are all involved in the metabolic process of APAP conversion to NAPQI. However, as reported by most researchers, CYP2E1 is responsible the largest part of APAP metabolism to NAPQI, while CYP1A2 and CYP3A4 contribute only little to this [25,26]. Meanwhile, several studies have reported that SM has only a little or no effect on the activity of CYP1A2 and CYP3A4 [27,28]. Collectedly, we focus on CYP2E1 in this study to investigate the effects of SM on APAP-induced liver injury. Our data showed that CYP2E1 expression was significantly upregulated after APAP overtreatment, that is consistent with other studies [29]. Upregulation of CYP2E1 expression can accelerate the production and accumulation of NAPQI. When the amount of NAPQI is excessive, the reduced GSH in the liver is consumed in large quantities. In addition, NAPQI binds to cysteine residues of proteins to form APAP-CYS, which leads to hepatic toxicity. Therefore, reducing the production of NAPQI can alleviate oxidative stress and prevent further hepatocytes death [30].

Our data indicated that SM pretreatment had no effect on glucuronide-mediated APAP metabolism in vivo and Cyp2e1 expression in normal rats and mice as mentioned earlier. However, we found that SM can significantly reduce the upregulated CYP2E1 mRNA and protein expression in ALI mouse liver, followed by decreased NAPQI, which results in the reduction of APAP-CYS and APAP-NAC in plasma, as well as the recovery of reduced GSH in the liver. Moreover, the AST and ALT values in mouse serum were decreased sharply in ALI mice pre-treated with series doses of SM compared with the solvent control group. The histological study of mouse liver also supported that SM pretreatment can notably alleviate liver pathological injury induced by APAP overdose in mice. Collectedly, we suggested that SM had benefits in the treatment of APAP-induced acute liver injury in the early stage via CYP2E1 inhibition. However, the specific mechanism of SM inhibition on Cyp2e1 expression in ALI state should be verified by further studies.

## 4. Materials and Methods

### 4.1. Materials

Chlorzoxazone (CLX, purity 98.0%), silymarin (SM, purity 97.5%), genistein (GEN, purity 98.0%), APAP (purity 99.0%), and hydroxypropyl β-cyclodextrin (HP-β-CD, purity 98.0%) were purchased from Dalian Meilun Biotechnology Corporation (Dalian, China). APAP-gluc (purity 98.0%), APAP-GSH (purity 95.0%), and APAP-CYS (purity 98.5%) were purchased from Toronto Research Chemicals (Toronto, ON, Canada). 6-OH-CLX (purity 95.0%) was purchased from GLPBIO Montclair (Montlcair, CA, USA). HLMs (Mixed Gender 50-Donor Pooled) and RLMs were purchased from Bioreclamation IVT (Baltimore, MD, USA). PMSF and heparin were purchased from Sigma-Aldrich (St. Louis, MO, USA).

### 4.2. Animals and Experimental Design

Male SD rats (aged 8–10 weeks and weighing 200–250 g) and male C57/BL6 mice (aged 6–8 weeks and weighing 20–25 g) were fed in 12 h of light and 12 h of darkness for 7 days in the animal housing facility of the West China School of Pharmacy, Sichuan University (Chengdu, China). The mice fasted for 12 h before the experiment and were freely provided drinking water. According to the equivalent dose coefficient conversion method of human and experimental animals, the maximum dose of SM for rats and mice was 45 mg/kg and 65 mg/kg, respectively. The equivalent dose conversion was based on guidance from the FDA (Guidance for Industry Estimating the Maximum Safe Starting Dose in Initial Clinical Trials for Therapeutics in Adult Healthy Volunteers, https://www.fda.gov/regulatory-information/search-fda-guidance-documents/estimating-maximum-safe-starting-dose-initial-clinical-trials-therapeuticsadult-healthy-volunteers, accessed on 6 December 2021).

SD rats were randomly divided into two groups (six rats in each group). One group was orally administered 4% HP-β-CD (*w*/*v*) solution as a solvent control group and the other group was orally administered SM 45 mg/kg (dissolved in 4% HP-β-CD (*w*/*v*)) for 21 consecutive days. On the 22nd day, rats in the two groups were given a single oral dose of CLX 30 mg/kg (suspended in 0.5% CMC-Na solution (*w*/*v*)). At 5 min, 10 min, 20 min, 30 min, 45 min, 1 h, 2 h, 3 h, 4 h, 6 h, 8 h, and 10 h after administration, blood samples (200 µL) were collected into centrifuge tubes containing 10 μL of heparin (1250 U/L). After the final blood collection, rats were euthanized by intraperitoneal injection of 50% urethane.

C57/BL6 mice were randomly divided into five groups (six mice in each group). The solvent control group and APAP group were orally administered 4% HP-β-CD (*w*/*v*), and the other three groups were orally administered SM 35/50/65 mg/kg (dissolved in 4% HP-β-CD (*w*/*v*)) for 21 consecutive days. On the 22nd day, the solvent control group was injected with saline intraperitoneally and the remaining group was injected with APAP (350 mg/kg). After APAP treatment for 6 h, blood samples were collected for APAP, APAP-gluc, APAP-GSH, and APAP-CYS measurement. After APAP treatment for 24 h, livers were harvested for qPCR, western blot, GSH measurement, and H&E analyses, and blood samples were collected for ALT and AST measurement.

This study was conducted according to the guidelines of the Declaration of Helsinki and approved by the Animal Ethics Committee of Sichuan University (No. K2022010).

### 4.3. In Vitro Metabolism Study

The probe substrate CLX, 50 mM potassium phosphate buffer (KPI, pH 7.4), and 0.25 mg/mL HLMS or RLMs were mixed and then an NADPH-regenerating system (1 mM β-NADP, 5 mM D-glucose-6-phosphate, 1 U/mL glucose-6-phosphate dehydrogenase, and 5 mM MgCl_2_) were added to initiate reaction. The HLMs/RLMs concentration and the incubation time selected in the in vitro metabolism system was according to the data shown in the Supplementary Materials, Figure S3. The total reaction system was 100 μL, in which the organic solvent content did not exceed 1%. HLMs or RLMs were incubated with CLX at 37 °C for 30 min. At the end of the incubation, 100 µL of precooled acetonitrile was added to terminate the reaction. After vortexing and centrifugation at 14,000 rpm for 5 min, 50 µL of supernatant mixed with 10 µL of the internal standard GEN was removed, and the sample was analyzed by HPLC–MS/MS.

The IC_50_ of silymarin on CYP2E1 was determined by incubating 0.25 mg/mL HLMs/RLMs with 300 µM CLX in the presence of a series of SM concentrations (0.5–200 μM) for 30 min at 37 °C. Activities were expressed as a percentage of 6-OH-CLX production in the blank control.

To assess the effect of SM on the general kinetics of CYP2E1 in microsomes, HLMs/RLMs with or without 20 μM SM were incubated with 6.25–1000 µM CLX for 30 min. Nonlinear regression analysis in GraphPad was used to calculate Km and Vmax. To determine the inhibitory type of CYP2E1 by SM, the above data were plotted as a Lineweaver–Burk plot.

To obtain the inhibition index (Ki) of SM on CYP2E1 in microsomes, 300/500 μM CLX with HLMs/RLMs was incubated with 1–200 µM silymarin at 37 °C for 30 min. Inhibition data were plotted as Dixon plots, and the inhibition constant (Ki) was calculated from the regression equation.

### 4.4. Sample Analysis

The concentrations of APAP and its metabolites in rat plasma and the concentrations of CLX and 6-OH-CLX in rat plasma, and the phase I metabolic system in vitro were determined by HPLC—MS/MS. CLX, 6-OH-CLX, APAP, APAP-gluc, APAP-GSH, APAP-CYS, and GEN (internal standard) were detected by multiple reaction monitoring of the *m*/*z* transitions 168.1-131.4, 184.2-119.7, 152.1-109.6, 328.1-152.3, 457.5-328.3, 271.2-139.9, and 269.5-133.0 for CLX, 6-OH-CLX, APAP, APAP-gluc, APAP-GSH, APAP-CYS, and GEN, respectively. Briefly, a 50 μL plasma sample was vortexed with 10 μL of the internal standard solution, and 150 μL pf methanol was then added to the mixture and shaken for 3 min to precipitate the protein. The sample was centrifuged at 14,000 rpm for 5 min, and 10 µL of the supernatant was analyzed by LC–MS/MS. The MS parameters and gradient elution of the tested samples are listed in Supplementary Tables S1 and S2. The noncompartmental analysis using Phoenix WinNonlin software (version 6.3, Pharsight Corp, Mountain View, CA, USA) was used to calculate the pharmacokinetic parameters of CLX and 6-OH-CLX.

The levels of ALT and AST in mouse serum and reduced GSH in mouse livers were measured by West China Frontier Pharmatech (Chengdu, China).

### 4.5. Cell Culture and Treatment

HepG2 human hepatoma cells were purchased from National Collection Authenticated Cell Cultures, Chinese Academy of Sciences (SCSP-510) and cultured at 37 °C in a humidified atmosphere containing 5% CO_2_. HepG2 cells were seeded into 96-well plates at a density of 1 × 10^4^ cells/well and 6-well plates at a density of 2 × 10^5^ cells/well. Cytotoxicity was assessed after incubation with a series of concentrations of SM for 24 and 48 h. To investigate the effect of SM on CYP2E1 expression, HepG2 cells were treated with nontoxic concentrations. After treatment with SM, total RNA and protein were extracted from HepG2 cells, then RT-qPCR and Western blotting were used to analyze the mRNA and protein expression levels.

### 4.6. RT-qPCR

Total RNA was isolated from HepG2 cells and frozen mouse liver tissues using TRIzol reagent (GBCBIO, Guangzhou, China) according to the manufacturer’s instructions. RNA concentrations were determined with a NanoDrop 2000 spectrophotometer (Thermo Fisher Scientific, Waltham, MA, USA) at 260 nm. RNA purity was determined with the absorbance at 260 nm versus 280 nm, with a ratio between 2.0 to 2.1. cDNA was synthesized from RNA (1 μg) using Hifair™ 1st strand cDNA Synthesis SuperMix (Yeasen Biological Technology Co. Ltd., Shanghai, China). Hieff™ qPCR SYBR^®^ Green Master Mix (Yeasen) was used to complete RT-qPCR. Fold changes in CYP2E1 expression in the drug-treatment groups compared with the blank control group were calculated using the 2^- ΔΔCT^ method with normalization to the internal control human GAPDH or mouse GAPDH. The primers were purchased from Tsingke Biological Technology (Chengdu, China) and the sequences of the specific primers are listed in Supplementary Table S3.

### 4.7. Western Blotting

Mouse liver tissue homogenates or HepG2 cells were mixed with RIPA lysis buffer, shaken, and centrifuged. The supernatant was used to prepare protein samples with 1× electrophoresis sample buffer. BCA protein assay kit (Biyuntian Co Ltd., Shanghai, China) was used to determine the content of protein. To separate protein samples, 12% SDS-PAGE was used and then protein was transferred onto a PVDF membrane. After washing with 5% skim milk to remove unbound proteins, the membrane was first incubated with rabbit anti-human/mouse CYP2E1 (1:1000 final dilution) and rabbit anti-human/mouse GAPDH (1:2000 final dilution) at 4 °C overnight, followed by 1 h incubation with secondary antibody (HRP Goat Anti-Rabbit IgG (H + L) used for CYP2E1 (1:1000 final dilution) and GAPDH (1:2000 final dilution)). Antibodies against CYP2E1 were kindly provided by Proteintech Biotechnology. ImageJ software version 1.53t (National Institutes of Health, Bethesda, MD, USA) was used to determine the densities of protein bands.

### 4.8. H&E Staining

The right lower lobe of the mouse liver was fixed with 4% paraformaldehyde for 24 h in preparation for H&E staining. The embedding and slicing processes for H&E staining were performed by Ao’Chuang Biotechnology Co., Ltd. (Chengdu, China). Images were acquired with a phase contrast microscope with 20× and 40× objective lenses. The scale bars were added with ImageJ software.

### 4.9. Statistical Analysis

The data are presented as the means ± SDs. IBM SPSS Statistics version 25 (IBM, Armonk, NY, USA) was used to complete the data analysis. Majority sets of quantitative data were analyzed by One-way ANOVA with Bonferroni’s multiple comparison test. All other data were analyzed by Student’s *t*-test. A value of *p* < 0.05 was considered to be statistically significant.

## 5. Conclusions

Our study showed that SM inhibited CYP2E1 enzyme activity in RLMs/HLMs and rats. In addition, SM downregulated the expression of CYP2E1 in HepG2 cells and inhibited the upregulation of Cyp2e1 expression in ALI mice. SM pretreatment reduced the levels of ALT, AST, and APAP-CYS and restored the hepatic level of GSH. These results suggest that SM protects against APAP-induced acute liver injury by reducing the activity and expression of CYP2E1.

## Figures and Tables

**Table 1:** Enzyme kinetic parameters of CLX in HLMs and RLMs.

	Control		Silymarin	
	V_max_ (nM/h/mg Protein)	K_m_ (μM)	V_max_ (nM/h/mg Protein)	K_m_ (μM)
HLMs	3.78 ± 0.34	397.2 ± 85.8	4.56 ± 1.67	1827 ± 318 **
RLMs	10.87 ± 1.92	619.6 ± 216.3	8.63 ± 2.43	1679 ± 413 *

* *p* < 0.05, ** *p* < 0.01 compared with the control group.

**Table 2:** Pharmacokinetic parameters of CLX in the solvent control and SM groups after a single oral dose of 30 mg/kg CLX.

PK Parameters	Control Group	Silymarin Group
*t*_1/2_ (h)	1.68 ± 0.73	3.92 ± 1.25 **
*t*_max_ (h)	0.75 ± 0.67	0.58 ± 0.30
*C*_max_ (ng/mL)	16,600.00 ± 4681.45	23,116.67 ± 6467.89
*V*_d_ (mL/kg)	1871.34 ± 1107.17	1566.26 ± 674.78
CL (mL/h/kg)	745.77 ± 266.34	272.12 ± 41.99 **
MRT (h)	2.56 ± 0.83	5.94 ± 1.63 **
AUC_0-t_ (h × ng/mL)	42,888.64 ± 14,453.20	91,847.01 ± 16,565.67 ***
AUC_0-∞_ (h × ng/mL)	44,469.95 ± 14,578.90	11,2307.50 ± 16,077.58 ***

** *p* < 0.01, *** *p* < 0.001 compared with the solvent control group.

**Table 3:** Pharmacokinetic parameters of 6-OH-CLX in the solvent control and SM groups after a single oral dose of 30 mg/kg CLX.

PK Parameters	Control Group	Silymarin Group
AUC_0-t_ (h × ng/mL)	287.58 ± 106.13	155.36 ± 28.45 *
AUC_0-∞_ (h × ng/mL)	325.78 ± 127.17	162.45 ± 22.18 *

* *p* < 0.05 compared with the solvent control group.

## Data Availability

The data underlying this article will be shared on reasonable request to the corresponding author.

## Supplementary Materials

The following are available online at https://www.mdpi.com/article/10.3390/molecules27248855/s1, Figure S1: The effect of silymarin on cell viability was determined by an MTT assay. Figure S2: Protein expression of CYP2E1 in rat liver after 21 days of silymarin pretreatment. Figure S3: Screening of the incubation time (A) and microsomal concentrations (B) in the in vitro metabolism system of HLMs and RLMs. Table S1: HPLC—MS/MS conditions for detection of CLX and 6-OH-CLX. Table S2: HPLC—MS/MS conditions for detection of APAP and its metabolites. Table S3: Primer sequences for real-time quantitative PCR (RT-qPCR). Table S4: Silymarin concentrations in plasma and liver of rats after multiple doses of silymarin administration.

## Sample Availability

Samples of the compounds silymarin are available from the authors.
