# Supplementary material for: Silymarin Protects against Acute Liver Injury Induced by Acetaminophen by Downregulating the Expression and Activity of the CYP2E1 Enzyme
Source: Molecules. 2022 Dec 13;27(24):8855. doi: 10.3390/molecules27248855 (PMC9784215; doi:10.3390/molecules27248855)
Supplement: Supplementary file 1 [file molecules-27-08855-s001.zip › molecules-2056142-supplementary.pdf]

Supplementary materials: Silymarin Protects Against Acute Liver Injury Induced by Acetaminophen via Downregulating the Expression and Activity of CYP2E1 Enzyme

Weipei Yang, Zhongxu Liang, Chengming Wen, Xuehua Jiang and Ling Wang

1. Figures

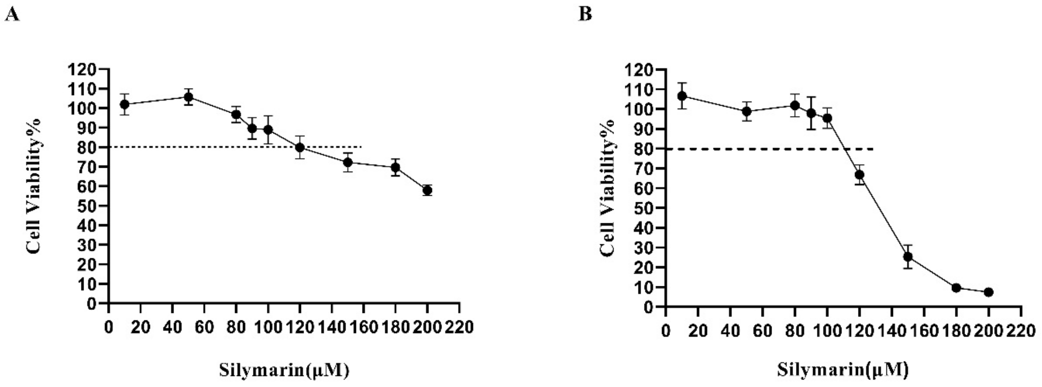

Figure S1. The effect of silymarin on cell viability was determined by an MTT assay. HepG2 cells were treated with different concentrations of silymarin for 24 h (A) and 48 h (B). Data were expressed as the mean ± SD, n=3.

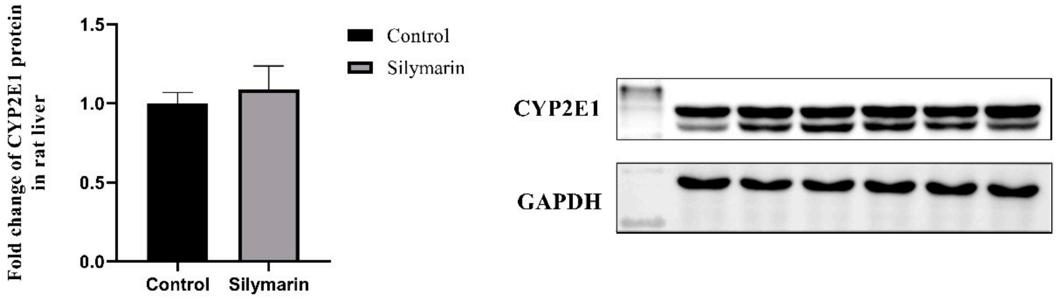

Figure S2. Protein expression of CYP2E1 in rat liver after 21 days of silymarin pretreatment. The protein expression levels of CYP2E1 were normalized to GAPDH. Data were expressed as mean ± SD, n=6.

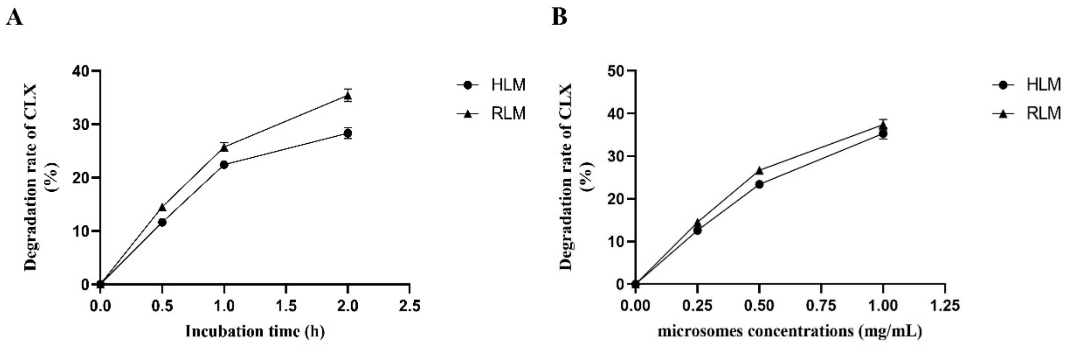

Figure S3. Screening of the incubation time (A) and microsomal concentrations (B) in the *in vitro* metabolism system of HLMs and RLMs. Data were expressed as the mean ± SD, n=3.

As most reportedly, the substrate conversion rate is generally limited between 10%-20% in the *in vitro* studies on CYP450 enzyme-mediated metabolism. Thus, we selected 0.25 mg/mL HLMs/RLMs incubated 0.5 h as the incubation condition in our study on the effects of SM on CYP2E1-mediated metabolism according to the data shown in Figure S3.

2. Tables

Table S1 HPLC–MS/MS conditions for detection of CLX and 6-OH-CLX

Analyte	Chlorzoxazone	6-OH-Chlorzoxazone
MRM transition (Q1-Q3 m/z)	168.1-131.4	184.2-119.7
Collision Energy	-34.2	-27.0
Declustering Potential	-36.7	-49.1
Chromatographic column	CAPCELL PAK C18, 50 × 2.00 mm 5um	
Mobile phase	A: 5mM ammonium acetate in 0.1% formic acid water<br>B: Methanol	
Gradient pattern	0-0.50 min/10% B, 0.51-2.00 min/95% B,<br>2.01-2.50 min/10% B	

Table S2 HPLC–MS/MS conditions for detection of APAP and its metabolites

Analyte	APAP	APAP-gluc	APAP-GSH	APAP-CYS
MRM transition (Q1-Q3 m/z)	152.1-109.6	328.1-152.3	457.5-328.3	271.2-139.9
Collision Energy	23.1	28.4	25.7	30.4
Declustering Potential	72.9	45.3	65.3	47.6
Chromatographic column	CAPCELL PAK C18, 50 × 2.00 mm 5um			
Mobile phase	A: 0.1% formic acid in water, B: Methanol			
Gradient pattern	0-1.00min/10% B, 1.01-2.50 min/90% B, 2.51-3.00 min/10% B			

Table S3 Primer sequences for real-time quantitative PCR (RT–qPCR)

Gene	Source	Forward (5' to 3')	Reverse (5' to 3')	Amplicon
CYP2E1	human	AATGGACCTACCT GGAAGGAC	CCTCTGGATCCGG CTCTCATT	AATGGACCTACCTGGAAGGACATCCGGCGGTTTTCCCTGACCACCCTCCGGAACTATG GGATGGGGAAACAGGGCAATGAGAGCCGGATCCAGAGG
GAPDH	human	CTGGGCTACACTG AGCACC	AAGTGGTCGTTG AGGGCAATG	CTGGGCTACACTGAGCACCAGGTGGTCTCCTCTGACTTCAACAGCGACACCCACTCC TCCACCTTTGACGCTGGGGCTGGCATTGCCCTCAACGACCACTT
Cyp2e1	mice	TTTCCCTAAGTATC CTCCGTGACT	GCTGGCCTTTGGT CTTTTTG	TTTCCCTAAGTATCCTCCGTGACTGGGGAATGGGGAAACAGGGTAATGAGGCCCGCAT CCAAAGAGAGGCACACTTCCTGGTGGAGGAGCTCAAAAAGACCAAAGGCCAGC
Gapdh	mice	AGGTCGGTGTGAA CGGATTTG	TGTAGACCATGTA GTTGAGGTCA	AGGTCGGTGTGAACGGATTTGGCCGTATTGGGCGCCTGGTCACCAGGGCTGCCATTT GCAGTGGCAAAGTGGAGATTGTTGCCATCAACGACCCCTTCATTGACCTCAACTACAT GGTCTACA

Table S4 Silymarin concentrations in plasma and liver of rats after multiple doses of silymarin administration (n=6)

	plasma	liver tissue
Concentration (μM)	0.35 ± 0.06	12.28 ± 1.06
